# *Lactobacillus* sp. participated in the adaptation of Rongchang piglets to cold stress

**DOI:** 10.17221/54/2023-VETMED

**Published:** 2023-10-23

**Authors:** Jie Chai, Xi Long, Pingxian Wu, Jinyong Wang, Xiaoqian Wu, Zhi Tu, Minghong Wei, Zongyi Guo, Tinghuan Zhang, Li Chen

**Affiliations:** ^1^Chongqing Academy of Animal Science, Chong Qing, Rongchang, P.R. China; ^2^National Center of Technology Innovation for Pigs, Chong Qing, Rongchang, P.R. China

**Keywords:** energy metabolism; gut microbiome, low temperature, production performance

## Abstract

Rongchang piglets were easily induced to cold stress and diarrhoea in the winter when raised in an open hog house. However, they also gradually recovered under mid-cold stress. Other studies have suggested gut microbiome might be involved in the host energy metabolism to relieve stress. To study how to adapt Rongchang piglets to cold stress by gut microbiome, thirty Rongchang piglets were randomly divided into a mild cold stress group and a control group for 30 consecutive days. The findings revealed that the piglets had low growth performance and a high diarrhoea rate and mortality rate during the first half of the cold treatment, but subsequently stabilised. The level of cortisol (COR) also displayed a similar trend. In the mild cold stress group, the relative abundance of *Muribaculaceae* significantly increased on day 15, and the predominant bacterial on day 30 was *Lactobacillus* sp. Our results indicated that the Rongchang piglet’s production performance and health were impaired at the start of the mild cold stress. However, as time passed, the body could progressively adapt to the low temperature, and *Lactobacillus* sp. participated in this process. This study provides new insight into how to alleviate health damage caused by cold stress.

Rongchang pigs are one of the top three pigs in China, and superior to European hybrid pigs (Zhang and Xiao 2004; Ma et al. 2015) having the characteristics of strong adaptability, tolerance to rough feeding, good meat quality, and stable genetic performance (Lai et al. 2007; Chen et al. 2020). However, the growth and development of piglets is not complete (Zeng et al. 2012), which makes them susceptible to temperature and humidity fluctuations in the breeding environment, and affects their later fattening period. At present, Rongchang piglets are often raised in an open hog house by free-range farmers (Figure 1B), where the temperature is easily changeable due to the environmental conditions, especially in the winter (Andrews et al. 2021; Kim et al. 2022). According to the tendency of the thermal neutral zone in breeding pigs (Ross et al. 2015), the lower critical temperature of piglets is the highest when in cold environment (He et al. 2018), hence, the low temperature not only leads to the slow growth of piglets, but also leads to various underlying diseases (Zhang et al. 2020; Yu et al. 2021; Chen et al. 2022).

**Figure 1:**
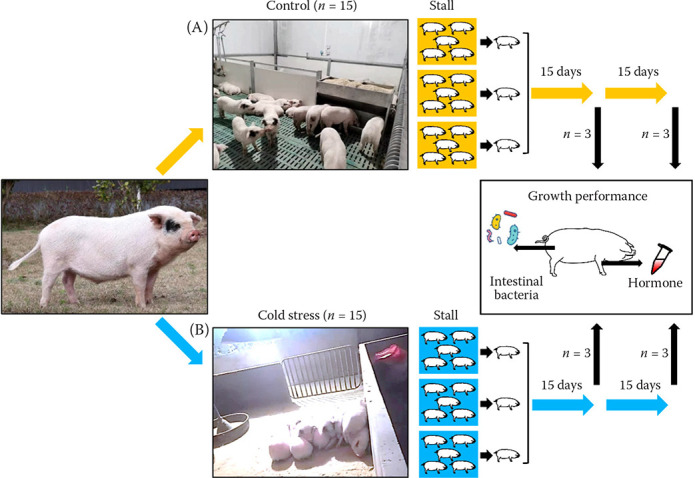
Experimental design and grouping

In husbandry, low temperatures can cause a stress response in an animal, that is, cold stress, which then affects the animal’s reproduction and growth (Browne et al. 2017; Yu et al. 2021; Zhang et al. 2022). The cold stress response is controlled by the hypothalamus-adenohypophysis-adrenal cortical axis, and the target glands relieve the adverse factors caused by stress through secreting cortisol (COR) (Xu et al. 2019). It helps the body to quickly adapt to the environment under mild cold stress.

In addition, at low temperatures, thyroid hormones and insulin (INS) can also increase the thermogenesis by regulating the energy metabolism (Hotamisligil and Davis 2016), which reduces feed utilisation. Low temperature can not only reduce the resistance of piglets, but can also cause intestinal damage and lead to diarrhoea in the piglets or their death. Some studies have proven that low temperature can cause oedema and dehydration of the intestine (Wilke et al. 2012; Chevalier et al. 2015) and microbiome disorders (Yang et al. 2018), which may be the reason of death in piglets. However, it is still unclear how Rongchang piglets can alter the abundance of gut microbiome and energy metabolism in order to promote the host adaptation to the stress environment under mild cold stress. In this experiment, we hypothesised that the piglets would initially be more sensitive to mild cold stress and this effect would be repaired and adapted through gut microbiota.

## MATERIAL AND METHODS

### Institutional review board statement

All the procedures performed in the study involving human participants were in accordance with the ethical standards of the institutional and national research committee and with the Helsinki declaration and its later amendments or comparable ethical standards. The study protocol was approved by Chongqing Academy of Animal Science’s Institutional Animal Care and Use Committee.

### Experimental design

According to the regulations of the laboratory animal protection and welfare of Chongqing Academy of Animal Science (XKY – No. 20220125), the 30 healthy Rongchang piglets (45 days old after being weaned for three days) with similar body weight were provided from the Rongchang District of Chongqing Municipality (105°30'E, 29°22'N). Their ears were individually labelled (Allflex, Drome, France) and randomly divided into a control group (*n* = 15, *n*_male_ = 7, *n*_female_ = 8, body mass = 11.44 ± 1.51 kg) and a mild cold stress group (*n* = 15, *n*_male_ = 8, *n*_female_ = 7, body mass = 11.99 ± 1.76) by a random number method.

**Figure 2:**
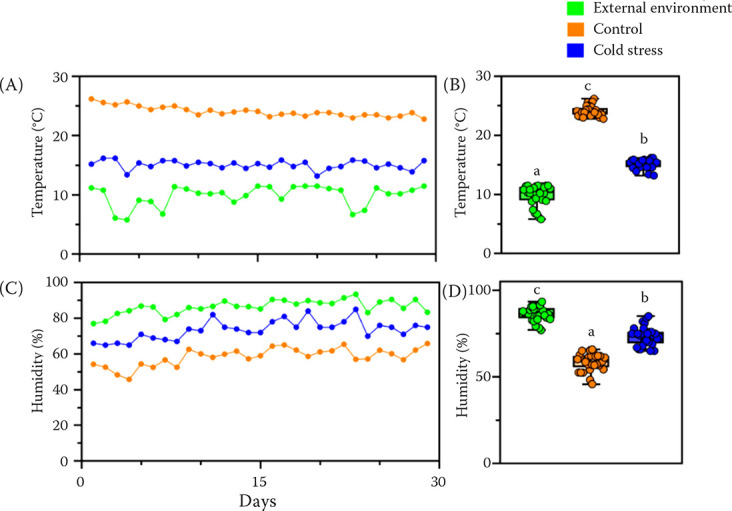
Changes in the temperature and humidity in the control group, the mild cold stress group, and the outdoor conditions (A) Line plots of the temperature changes over a 30-day period. (B) Significance analysis of the temperature change. (C) Line chart of the humidity changes over a 30-day period. (D) Significance analysis of the humidity change

Based on the preliminary test results, the control group was bred in a hog house where the ambient temperature could be controlled, and the mild cold stress group was bred in a traditional hog house for 30 days (from December 2021 to January 2022). To facilitate follow testing, the groups was then randomly divided into three stalls with five piglets in each stall (Figure 1). All the piglets were fed and could drink freely; the 951S suckling pig feed was purchased from Chongqing Zhengda Feed Co., Ltd. (Chongqing, P.R. China). Except for the temperature and humidity, the feed, light, personnel, feeding time, and other breeding environments were consistent (Figure 1). The average temperature and humidity of the two groups and the outdoor conditions were recorded for 30 days. After analysis, there were significant differences in these indices (Figure 2B,D), which indicated that the grouping was reasonable. At the same time, blood and fresh faeces were collected on days 15 and 30 for a dynamic analysis of the physiological indices of the Rongchang piglets.

### Measurement of the growth performance of the Rongchang piglets

The body weight of each piglet was measured on the 1^st^ and 30^th^ days of the experiment. The daily feed intake, diarrhoea, and health status of each piglet were recorded, and the average daily gain, average daily feed intake, feed-to-gain ratio (Equation 1), diarrhoea rate (Equation 2), and survival rate were collected from all the stalls and calculated by the following equations:

$$\text{Feed-to-gain ratio}=\frac{\text{Average daily feed intake}}{\text{Average daily gain}\times \text{Number of piglets}}$$ (1)

$$\text{Diarhhoea rate}=\frac{\text{Number of diarrhoea}\times \text{Days of diarrhoea}}{\text{Number}\times \text{Test days}}$$ (2)

### ELISA: Determination of the hormone levels in the Rongchang piglets

The concentration of cortisol was used to assess the stress levels of the Rongchang piglets. The growth and energy metabolism were evaluated by leptin, growth hormone (GH), T3, T4, and insulin (Beijing Northern Institute of Biotechnology Co., Ltd., Beijing, P.R. China). All the indicators were detected by an enzyme linked immunosorbent assay (ELISA) kit. The process was as follows: the blood samples of the Rongchang piglets were collected from the anterior cavity venous in a 5 ml sterile syringe (Shandong Aosite Medical Equipment Co., Ltd., Shandong, P.R. China) on 15 days and 30 days of the control and cold stress group. After allowing the blood samples to gain room temperature for 2 h and centrifuging at 3 500 rev/min for 10 min (5810; Eppendorf, Hamburg, Germany), we obtained the supernatant samples. A 50 μl sample and a 50 μl sample analysis buffer were mixed and incubated at room temperature for 120 min in a well, then 100 μl of biotinylated antibodies were added and was incubated for 60 min, 100 μl of horseradish peroxidase labelling was added and was incubated for 60 min, and 100 μl of a colour developing agent was added and was incubated for 20 min, finally 500 μl of a stop buffer was added and optical density of each sample at 450 nm was measured through a microplate reader (Bio-Rad, Hercules, CA, USA).

### 16S rRNA: Analysis of the gut microbes in the Rongchang piglets

The faeces of Rongchang piglets (2 groups × 2 periods × 3 stalls = 12 samples) were collected on the 15^th^ and 30^th^ days of the experiment and stored at –80 °C. After being collected, the faeces were tested by Novogene Co., Ltd. (Beijing, P.R. China). The specific process was as follows: The total faecal bacterial DNA was extracted using a DNA Isolation Kit (Qiagen, Hilden, Germany) following the manufacturer’s protocol. The V3–V4 regions of the bacterial 16S rRNA gene were amplified by polymerase chain reaction (PCR, 98 °C for 2 min, followed by 30 cycles of 98 °C for 30 s, 50 °C for 30 s, 72 °C for 60 s, and 72 °C for 5 min) using the primers 338F (5'-ACTCCTACGGGAGGCAGCAG-3') and 806R (5'-GGACTACHVGGGTWTCTAAT-3') (Huang et al. 2022). The PCR amplification products were tested quantitatively using the quantifiable Fluor^TM^-ST Blue fluorescence quantification system (Promega), proportioned Fluor^TM^-ST based on the sequencing volume requirements, and the libraries were constructed using the NEBNext^®^ Ultra^TM^ IIDNA Library Prep Kit (Nanjing WARBIO Co., Ltd., Jiangsu, P.R. China). The raw Fastq data were demultiplexed based on their barcodes, and the paired-end reads for all the samples were run through Trimmomatic (v0.35) to remove the low-quality base pairs. FLASH (v1.2.7) was used to join the paired reads and sequences over a 50-bp sliding window. Sequences with an average base quality score lower than 20 were removed, and overlapping lengths (> 10 bp) were retained. The demultiplexed reads were clustered with a 97% similarity cutoff using UPARSE (v10.0) to generate operational taxonomic units (OTUs). The taxonomic classification of the representative OTU sequences was undertaken using the Silva 138.1 database (confidence threshold: 0.6). The α diversity was analysed by the Chao index, Simpson index, and Shannon index, and the results showed that the α diversity was evenly distributed within the same group. The β diversity was analysed by a weighted UniFrac distance matrix and principal coordinate analysis (PCoA). The target bacteria with the greatest richness and the most significant changes were selected by *t*-test.

### Statistical analysis

All the measurements were set up in three replicates, and the data results for all the groups were the mean ± SD. An analysis of variance (ANOVA) and a *t*-test were used to analyse the data and plot them using GraphPad Prism v8; GraphPad Software Inc., San Diego, CA, USA), with *P* < 0.05 as the threshold of significance.

## RESULTS

### Mild cold stress-induced slow growth and diarrhoea in the Rongchang piglets

**Figure 3:**
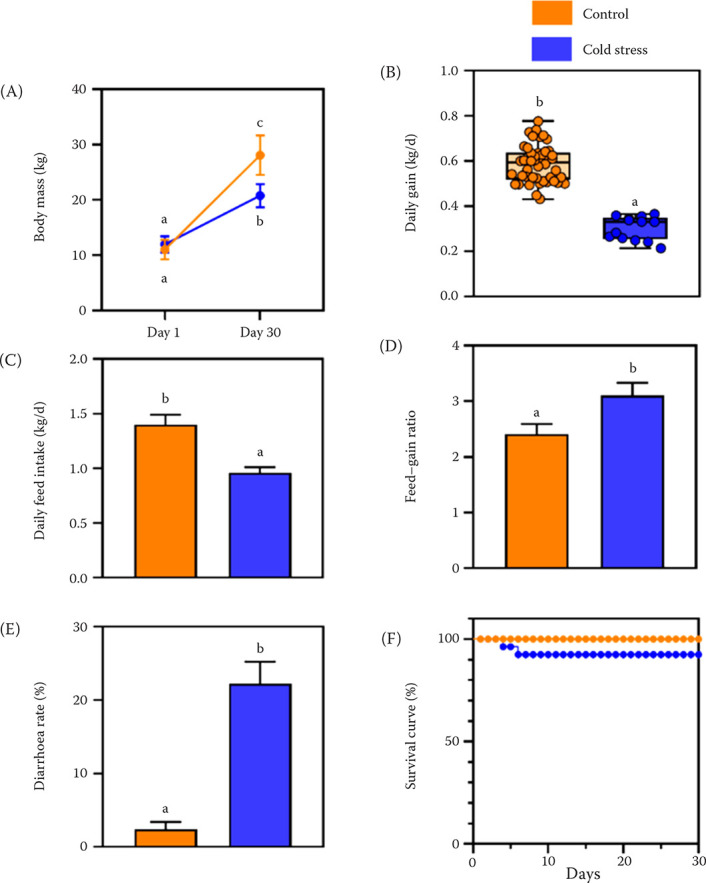
Effects of the cold stress on the growth performance of the Rongchang piglets Effects of the low temperature on the body weight (A); daily gain (B); daily food intake (C); feed–gain ratio (D); diarrhoea rate (E); survival rate (F) of Rongchang piglets

The effects of the cold stress on the growth performance of Rongchang piglets are very adverse. Compared with the control group, the body weight of piglets in the cold stress group was significantly decreased by 26.06% (Figure 3A; *P* < 0.05), and the daily gain and daily feed intake of the cold stress group were also lower than in the control group (Figure 3B,C; *P* < 0.05). Correspondingly, the feed-to-gain ratio of the piglets in the cold stress group was significantly increased by 28.63% (Figure 3D; *P* < 0.05). In addition, the diarrhoea rate in the cold stress group was higher than the control group (Figure 3E; *P* < 0.05), and the survival rate decreased after 4 and 6 days, then remained unchanged (Figure 3F; *P* < 0.05). Therefore, we can preliminarily judge that the decrease in feed utilisation and the increase in diarrhoea caused by the low temperature were the key reasons for the slow weight gain and increased mortality of the piglets.

### Rongchang piglets gradually adapt to mild cold stress

**Figure 4:**
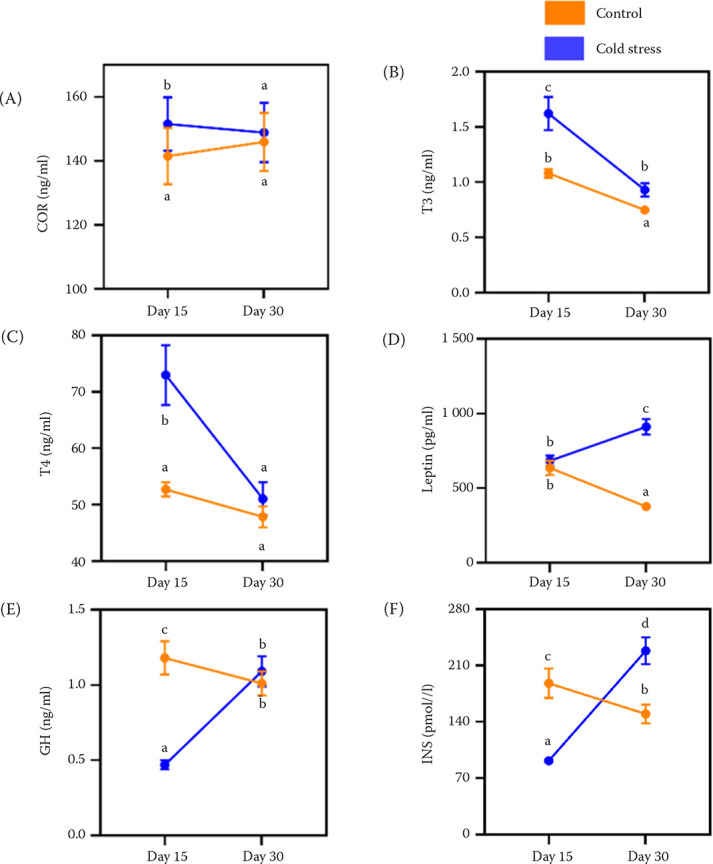
Effects of the cold stress on the energy metabolism of the Rongchang piglets Effects of the low temperature on the Rongchang piglets in cortisol (COR) (A), T3 (B), T4 (C), leptin (D), growth hormone (GH) (E), insulin (INS) (F)

Hormones are one of the important components of humoral regulation. The cold stress was mediated by a variety of hormones and affected the growth and energy metabolism. We found that cortisol in the cold stress group was significantly increased by 7% on day 15, then decreased (Figure 4A; *P* < 0.05).

T3 and T4 also showed the same trend and were significantly higher than the control group on day 15 (Figures 4B,C; *P* < 0.05). From 15 to 30 days after the cold treatment, leptin, growth hormone and insulin showed an increasing trend, compared with the control group (Figure 4D–F; *P* < 0.05).

These results indicated that the stress response of Rongchang piglets changed significantly in the early stages of the cold treatment, and then recovered gradually.

### Effects of the cold stress on the gut microbiome of Rongchang piglets

**Figure 5:**
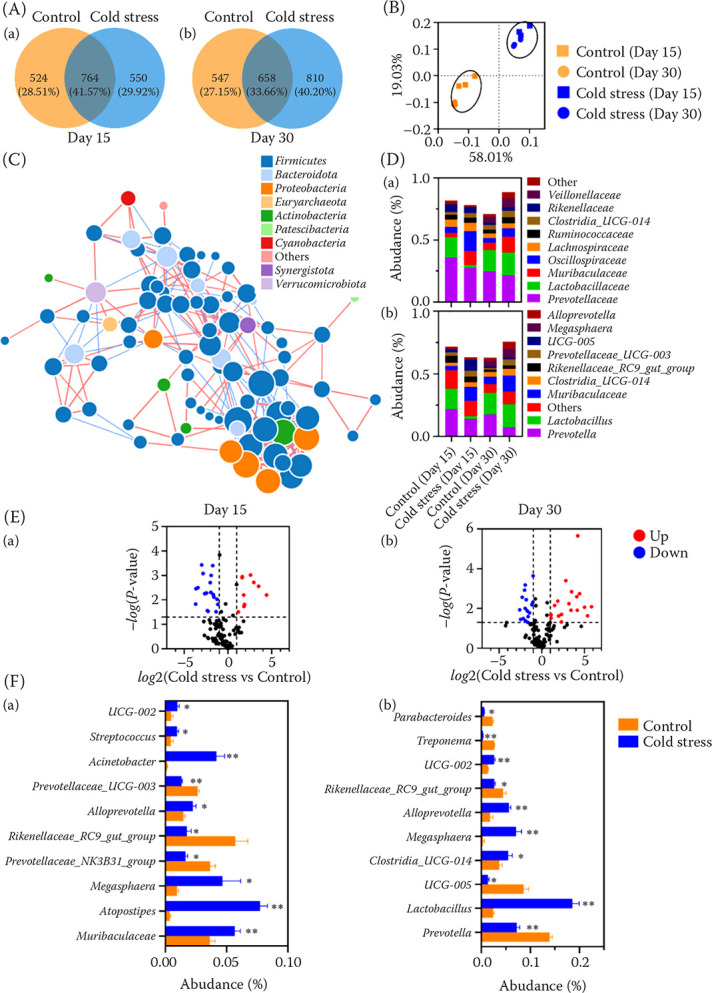
Effects of the cold stress on the gut microbiome of Rongchang piglets (A) Venn map for the groups on day 15 (a) and day 30 (b). (B) PCoA analysis for the groups on day 15 and day 30. (C) Network map at the phylum level. (D) Relative abundance of the top 10 bacteria at the family (a) and genus (b) levels. (E) Volcanic map on day 15 (a) and day 30 (b) at the genus levels. (F) Significant analysis of the major bacteria at the genus level on days 15 (a) and 30 (b)

The gut bacteria affect the host energy metabolism and gut health. An analysis found differences between the control group and the cold stress group (Figure 5B), despite 41.57% and 32.66% common operational taxonomic units (OTUs) at days 15 and 30, respectively (Figure 5A).

The network map also showed that Firmicutes was the dominant bacteria in the Phylum level (Figure 5C), which was most likely related to the effects of the cold stress and the balance of the intestinal bacteria.

An analysis of the bacterial composition showed that the species of the bacteria in each group were the same (Figure 5Da,b), but the change multiples were significantly different (Figure 5Ea,b). At the genus level, the analysis of the top 10 bacteria with richness showed that the relative abundance of *Muribaculaceae* was the largest on the 15^th^ day of cold stress, with a significant increase of 55% (Figure 5Fa), while the relative abundance of *Lactobacillus* sp. was significantly increased by 6.78 times on the 30^th^ day (Figure 5Fb).

This provided a foundation to investigate the dynamic relationship between the cold stress and the gut microbiome.

## DISCUSSION

This study confirmed that the mild cold stress caused slower growth, decreased the feed conversion rate, and diarrhoea in Rongchang piglets. The cortisol increased significantly in the first half of the cold treatment, then adapted. The *Muribaculaceae* and *Lactobacillus* sp. were the dominant bacteria at days 15 and 30, respectively. These results indicated that mild cold stress can reduce the growth performance of Rongchang piglets and can be gradually adapted, which provides the possibility for studying healthy growth and development of Rongchang piglets in winter.

The focus of this experiment was on the growth performance of piglets and the factors that led to them decline. Simultaneously, we attempted to solve the contradiction between the growth and stress response by the gut-dominant microbiome. As endothermic animals, livestock need to increase their basal metabolism to offset the negative effects of low temperatures (Lees et al. 2019), resulting in slow weight gain and decreasing feed conversion. For low temperatures, animals often use two ways to maintain body temperature: one is to reduce heat dissipation, such as to huddle together for warmth to ensure that the temperature in the central location is warmer (Glancy et al. 2015), and the other is to increase heat production, such as using cortisol (COR) to promote skeletal muscle tremors (Jellyman et al. 2012), T3 and T4 to increase intracellular oxidation through Na^+^-K^+^ pumps on the cell membranes (Chahardoli et al. 2019), and insulin (INS) to promote the breakdown of carbohydrates to produce more heat (Dumke et al. 2015; Seibert et al. 2018). In this study, it was suggested that the expressions of COR, T3 and T4 increased first and then decreased with the extension of the mild cold stress time, which may be due to the gradual adaptation of Rongchang piglets to low temperature environments. So, the mortality and diarrhoea also occurred in the first half of the cold treatment.

The decrease in the daily feed intake of piglets may be due to leptin. Leptin, a hormone secreted by white adipose tissue (Zhang and Chua 2017), could increase in non-starvation and inhibit foraging through receptors in the central nervous system (Perry et al. 2018; Pereira et al. 2021). In addition, we also found that cold stress led to the recovery of the serum growth hormone (GH) levels in the Rongchang piglets after a significant decrease in the first half of the cold stress. The fact might be that the secretion of GH was regulated by the pituitary gland, and cold stress leads to the activation of the hypothalamic-pituitary-thyroid axis (Lal and Hoffman 2019), making its main physiological response change from body growth to increased body thermogenesis, and resulting in the synthesis and release of GH disorders, while the body gradually adapts to the low temperature environment in the second half of experiment. As a result, it can be concluded that the piglets were regulated by a variety of hormones in response to the mild cold stress, and the overall stress responded first by aggravating the studied factors and then by gradually adapting them.

Diarrhoea is related to the balance of the gut microbiome (Li et al. 2021). In this study, it was suggested that the dominant bacteria on the 15^th^ and 30^th^ days of cold treatment were *Muribaculaceae* and *Lactobacillus* sp. respectively in the genus level. Studies have shown that the outer structure of *Muribaculaceae* included bacteroid outer membrane vesicles (OMVs), and that OMVs can become pathogenic carriers, such as long-distance storage and transport of virulence factors (Lagkouvardos et al. 2019; Lieberman 2022), or participate in the catabolism of proteins to produce enterotoxins (Smith et al. 2021), which, in turn, leads to the decline in the host intestinal immunity or inflammation (Zhao et al. 2022). However, the *Lactobacillus* sp. abundance corresponds to the body recovery process. On the one hand, *Lactobacillus* sp. can improve the living environment of potential probiotics and inhibit the growth of harmful bacteria in the intestinal tract by secreting lactic acid (Slattery et al. 2019; Scillato et al. 2021). On the other hand, it can also secrete digestive enzymes (Dec et al. 2020; Mann et al. 2021), which was conducive to decomposing substances and improving the utilisation rate of the feed (Mukai et al. 2003). Combined with the changes in stress level of Rongchang piglets, we concluded that *Lactobacillus* sp. may alleviate stress response and is involved in cold adaptation through the above mechanisms.

In conclusion, the mild cold stress had a negative impact on the growth and health of Rongchang piglets, but can be gradually adapted and adjusted. In this process, *Lactobacillus* sp. played a key role as a part of the external environment providing a new idea for how to alleviate the damage caused by cold stress in the breeding process.
